# Antibiotic prescription after tooth extraction in adults: a retrospective cohort study in Austria

**DOI:** 10.1186/s12903-022-02556-w

**Published:** 2022-11-20

**Authors:** Safoura Sheikh Rezaei, Brigitte Litschauer, Karolina Anderle, Stephanie Maurer, Patrick Jan Beyers, Berthold Reichardt, Michael Wolzt

**Affiliations:** 1 Department of Clinical Pharmacology, Medical University of Vienna, Spitalgasse 13, 1090 Vienna, Austria; 2 Austrian Social Health Insurance Fund, Österreichische Gesundheitskasse, 7000 Eisenstadt, Austria

**Keywords:** Antibiotic prophylaxis, Cohort studies, Dentistry, Endocarditis prevention & control

## Abstract

**Background:**

Broad spectrum antibiotics are often used for the prophylaxis of infectious endocarditis and treatment of odontogenic infections, but there are limited data related to antibiotic use and adherence to prescription guidelines.

**Methods:**

Data from patients with tooth extraction between 2014 and 2018 were selected from a database of a regional health insurance fund. We created three data sets, one based on all tooth extractions, one on multiple teeth extractions, and one including only single tooth extraction. After data collection, descriptive analysis was carried out. The differences in prescription pattern of antibiotic medicine were tested by χ^2^ test, Student´s t-test or ANOVA.

**Results:**

From 43,863 patients with tooth extraction, 53% were female, and 3,983 patients (9.1%) filled a prescription for antibiotic medicine. From 43,863 patients, 157 patients (0.4%) had endocarditis risk, but only 8 patients of these (5.1%) filled an antibiotic prescription. In total, 9,234 patients had multiple and 34,437 patients had only one tooth extraction. Patients with more than one tooth extraction received more often antibiotic treatment (10.7%) compared to those with single tooth extractions (χ^2^ = 36; *p* < 0,001). Patients with more than one tooth extraction were older, however, younger patients received antibiotics more frequently (t = 28,774, *p* = 0.001). There was no relationship with endocarditis risk status. Clindamycin and amoxicillin/clavulanic acid were the most frequently prescribed antibiotic medicines.

**Conclusions:**

In this retrospective cohort study, dentists did not discriminate prophylactic antibiotic prescription with regard to endocarditis risk status. A factor influencing prescribing behaviour of antibiotic medicines was the number of extracted teeth.

## Background

Dentists prescribe around 10% of all prescribed antibiotic medicine in primary care [1]. Antibiotics are often prescribed as a prophylactic measure, in particular after tooth extraction [2]. Broad-spectrum antibiotics such as penicillin with or without clavulanic acid are the most commonly prescribed antibiotic treatments by dentist [3–7]. For patients with penicillin allergy clindamycin is a frequently used therapeutic alternative [8]. Antibiotic prescription after surgical procedures is also common in patients at risk to develop osteonecrosis of the jaw, which is linked to bisphosphonate and to denosumab treatment [9–12].

Antibiotic resistance is increasing and becoming a major healthcare problem [13]. Due to enhanced antibiotic prescription a corresponding antimicrobial resistance pattern has been observed [14]. Prophylactic antibiotic use in dentistry has been repeatedly addressed, as this may contribute to development of antibiotic resistance [15–17]. In comparison with other European countries, the use of antibiotics in Austria is only moderate [18]. Between the years 2011 and 2015, the total consumption of antibiotics has increased by 3.8%, with beta lactams being the preferred treatment in Austria [19]. The increase in antibiotic consumption continues, however, some countries have managed to reduce antibiotic prescription in recent years [20–22].

The clinical indication for antibiotic use in dentistry is controversially discussed in the literature. Dental extractions are performed for various reasons e.g. because of tooth caries, periodontitis, or impacted wisdom teeth. According to current guidelines only a small number of patients potentially benefits from antibiotic prescription for the prevention of infective endocarditis following invasive dental procedures [23, 24]. Some studies have reported a significant reduction in postoperative complications [25–27] while others [28, 29] did not find a positive outcome of routine use of antibiotic medicines. In particular, prophylactic antibiotic therapy after tooth extraction is under debate, since most of periodontal and dental infectious complications can be managed with simple oral hygiene measures or in urgent cases by surgical interventions.

The aim of the study was therefore to assess the practice of antibiotic prescription by dentists after tooth extraction in Austria. We also investigated the association of extraction procedure and/or number of teeth removed with antibiotic prescription patterns.

## Material and methods

This retrospective study was approved by the Ethics Committee of the Medical University of Vienna (EK-No. 1932/2019) and performed in accordance with the Declaration of Helsinki. Patients informed consent was not required due to the retrospective design of the present study. The Ethics committee waived the requirement of informed consent for the use of retrospective data, consistent with provisions of the data protection act related to research use of existing individual medical information. Data were pseudonymized in accordance with data protection provisions and transmitted electronically on a password-protected computer server. Data user restriction was applied to the first and corresponding author and the statisticians had access to the data.

This population-based cohort study was conducted in patients with a tooth extraction between 2014 and 2018 by using the database of the regional health insurance (Burgenländische Gebietskrankenkasse, BGKK). Demographic data as well as tooth extraction diagnosis codes according to International Classification of Diagnosis (ICD–10) [30], and information on single medical interventions and services (MEL) [31] were used. Patients with endocarditis risk were classified by using MEL and ICD codes (MEL: valve replacement (DB), reconstruction of aorta with or without valve replacement (DG); ICD: congenital malformation of heart cavities and valves (Q20, Q21, Q22, Q24), endocarditis (I33, I38)) for mechanical or biological heart valve replacement, history of endocarditis, and patients with an underlying heart disease and as well as heart transplant patients who developed a valve disease.

Also, data on prescribed medication by a dentist between extraction day (day 0) and three days after intervention was retrieved from the respective databases utilizing the Anatomical Therapeutic Chemicals (ATC) classification system. The ATC classification is an international classification for pharmacological agents. Substances are listed in different groups according to organs they affect and their chemical, pharmacological and therapeutic characteristics. In Austria each medication has an additional unique Austrian pharmaceutical registration number, which is linked to the ATC-codes [32]. Patients with antibiotic, antiphlogistic, analgesic, bisphosphate, and corticosteroid prescriptions were identified by ATC codes J01, M01, N01, M05BA and H02 and their subcodes, respectively. Bisphosphonate use was coded yes when prescribed within the last 6 months before the index event. Dental specialist/institution were either Tooth/Mouth/Maxillo-surgeon (TMM-SURGEON), dental practitioner (dentists), or TMM-SURGEON/dentists in out-patient clinics (DOC).

Data retrieval and collection into the databases and review were done by Berthold Reichardt, ÖGK. Data were pseudonymized before transfer and further storage and handling was also in accordance with data protection provisions.

### Cohort selection

**Fig. 1:**
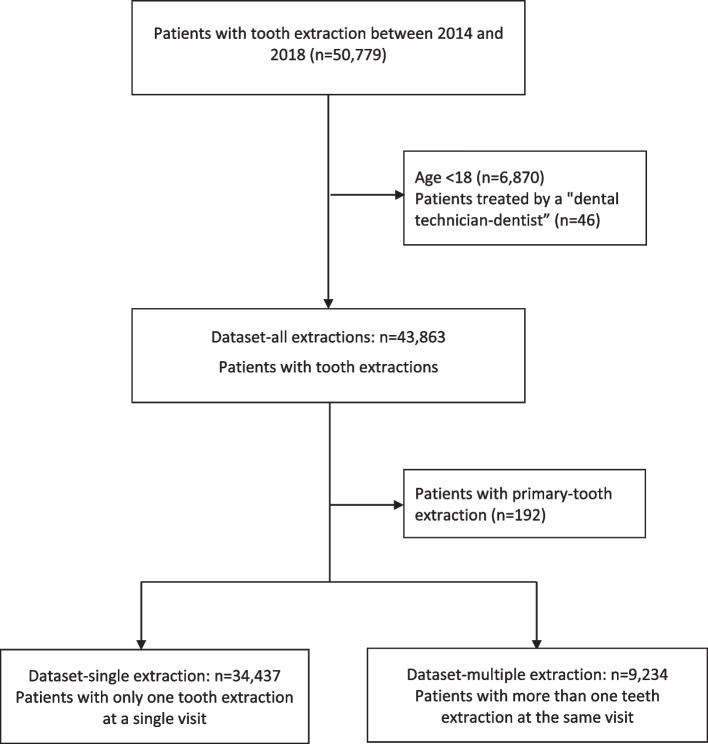
Flowchart

Data from 50,779 patients were eligible for our study (Fig. 1). 6,870 patients were younger than 18 years old and therefore not used for further analysis. In addition, a small percentage of 0,1% of patients (*n* = 46) were treated by a “dental technician-dentist” and excluded. During the observation period from 2014 to 2018 over 110,000 teeth were extracted in 43,863 adult patients. The number of teeth extracted ranged between one and 30 per patient within the observation period and between one and 25 per patient per day.

We therefore created three data sets, one based on all tooth extractions (dataset-all extractions, *n* = 43,863), one based on multiple teeth extractions (dataset-multiple extractions, *n* = 9,234), and one based on only single tooth extraction at the first visit (dataset-single-extractions, *n* = 34,437). For all datasets the first treatment was considered as the index event. The date of index event was used as reference for matching variables such as age, endocarditis risk, bisphosphonate intake, and filled antibiotic prescriptions.

The analysis set of dataset-all extractions therefore contains 43,863 patients with the following variables: age, sex, antibiotic and bisphosphonate prescription, endocarditis risk, and information on dental specialist/institution where the extraction was executed. The dataset-single extraction is limited to 34,437 patients with only one tooth extraction during the index intervention.

Tooth extraction was defined as three different types of procedures: simple extraction, surgical extraction, or retained tooth extraction. Tooth groups were categorized to form five categories (Incisors, canines, Premolars, 1^st^ and 2^nd^ molars, and 3^rd^ molars).

### Statistical analysis

A descriptive analysis of all available variables was performed. Continuous and categorical variables were expressed as mean ± standard deviation (SD) or frequencies and percent, respectively. Differences in antibiotic prescription rate for categorical variables were tested by χ2 test and for continuous variables by student's t- test or ANOVA. P values < 0.05 were regarded as statistically significant. For statistical analyses SPSS 11.0 for Windows (SPSS 11.0, SPSS Inc.) was used.

## Results

### Demographic data for dataset-all extractions (*n* = 43,863)

Table 1 presents the patients characteristics from dataset-all extractions. Bisphosphonates were prescribed in 1,005 patients (2,3%), from which 77 patients (7.7%) had a prescription of antibiotics. 157 patients (0.4%) had an endocarditis risk, from which 8 patients (5.1%) filled a prescription of antibiotic medicine. The treatment of patients with endocarditis risk or the prescription of bisphosphonate were not associated with treating specialist/institution. Tooth extractions of outpatients were done by TMM-SURGEON in 34,429 patients (78.5%), followed by dentists in 6,544 patients (14,9%), and by out-patient clinics in 2,890 patients (6,6%).

**Table 1:** Patient demographics

		**All extractions (*n* = 43,863)**	
		n	%
**Sex**	female	23,334	53.2
	male	20,529	46.8
**Endocarditis risk**	no	43,706	99.6
	yes	157	0.4
**Bisphosphonate prescription**	no	42,858	97.7
	yes	1,005	2.3
**Type of dental institution**	TMM-SURGEON	34,429	78.5
	dentists	6,544	14.9
	DOC	2,890	6.6

*TMM-SURGEON* Tooth/Mouth/Maxillo-surgeon, *dentists* dental practitioner, or *DOC* TMM-SURGEON/dentists in out-patient clinics

### Demographic data for multiple teeth extraction (*n* = 9,234)

Bisphosphonates were prescribed in 271 patients (2.9%) and 49 patients (0,5%) had an endocarditis risk. Tooth extraction of outpatients were done by TMM-SURGEON in 78.5%, followed by dentists in 14.9%, and by and out-patient clinics in 6.6% of patients.

### Demographic data for single-tooth extraction (*n* = 34,437)

From 34,437 patients from dataset-single extraction, 18,405 female and 16,032 male patients with an age range of 18–99 years were analyzed. 108 patients (0.3%) had an endocarditis risk and 734 patients (2.1%) took bisphosphonates.

### Antibiotic prescription

Table 2 presents antibiotic prescription from dataset single extraction and all extraction. Patients with more than one tooth extraction received significantly more often antibiotic treatment compared to those with single tooth extractions (10.7% vs.8,6%; χ^2^ = 36; *p* < 0.001). Although patients with more than one tooth extraction were older, patients with antibiotic prescription were significantly younger (46 ± 17 vs. 54 ± 18; t = 28,774; *p* = 0.001).

**Table 2:** Antibiotic prescription

		**All extractions** **(*n* = 43,863)**		**Single tooth extractions** **(*n* = 34,437)**	
		*n* =	%	*n* =	%
**Antibiotic medicine (ATC-Code: J01)**	no	39,880	90.9	31,445	91.3
	yes	3,983	9.1	2,992	8.7
**Type of Antibiotic**	Clindamycin (J01FF01)	2,012	50.5	1,288	43.0
	Amoxicillin/clavulanic acid (J01CR02)	1,658	41.6	1,461	48.8
	others	313	7.9	243	8.1

Antibiotic prescription presented according to different antibiotic types and extraction group

Clindamycin (J01FF01) and amoxicillin/clavulanic acid (J01CR02) were the most frequently prescribed antibiotic medicine. As shown in Table 3, dentists prescribed more often antibiotics (11.8/11.1%) compared to TMM-SURGEON (8.8/8.5%) and DOC (5.5/5.3%) (χ2 = 108/66; *p* < 0.001). A similar significant difference in prescription rate was also shown for amoxicillin/clavulanic acid compared to clindamycin and other antibiotics in database-all-extractions (χ^2^ = 310; *p* < 0.001) and in database-single-extractions (χ^2^ = 226; *p* < 0.001) (Table 4).

**Table 3:** Antibiotic prescription according to specialist

		All tooth extractions		Single tooth extractions	
		no	yes	no	yes
TMM-SURGEON	n (%)	31,380 (91.1%)	3,049 (8.9%)	25,167 (91.5%)	2,339 (8.5%)
Dentist	n (%)	5,769 (88.2%)	775 (11.8%)^a^	4,404 (88.9%)	549 (11.1%)^a^
DOC	n (%)	2,731 (94.5%)	159 (5.5%)	1,874 (94.7%)	104 (5.3%)
Total	n (%)	39,880 (90.9%)	3,983 (9.1%)	31,445 (91.3%)	2,992 (8.7%)
		χ^2^ = 108; *p* < 0.001		χ^2^ = 66; *p* < 0.001	

^a^indicates significance; *TMM-SURGEON *Tooth/Mouth/Maxillo-surgeon, *dentists* dental practitioner, or *DOC* TMM-SURGEON/dentists in out-patient clinics

**Table 4:** Antibiotic prescription according to medicine and specialist

		**All tooth extractions**			**Single tooth extractions**		
		clindamycin	amoxi/clav^a^	others	clindamycin	amoxi/clav^a^	others
TMM-SURGEON	n (%)	1,486 (48.7%)	1,378 (45.2%)	185 (6.1%)	1,163 (49.7%)	1,028 (44.0%)	148 (6.3%)
Dentists	n (%)	135 (17.4%)	518 (66.8%)	122 (15.7%)	105 (19.1%)	353 (64.3%)	91 (16.6%)
DOC	n (%)	37 (23.3%)	116 (73.0%)	6 (3.8%)	20 (19.2%)	80 (76.9%)	4 (3.8%)
Total	n (%)	1,658 (41.6%)	2,012 (50.5%)	313 (7.9%)	1,288 (43.0%)	1,461 (48.8%)	243 (8.1%)
		χ^2^ = 310; p < 0.001			χ^2^ = 226; p < 0.001		

^a^indicates significance; *TMM-SURGEON* Tooth/Mouth/Maxillo-surgeon, *dentists* dental practitioner , or  *DOC* TMM-SURGEON/dentists in out-patient clinics, amoxi/clav, Amoxicillin/clavulanic acid

### Extraction and procedure type

For this analysis we used the dataset-single tooth extraction. Most single-tooth extraction procedures were done as simple extraction (54.6%) followed by surgical extraction (41.2%) and retained tooth extraction (4.2%). Tooth extraction details are presented in Table 5. More than 60% of all extractions were carried out in molar teeth.

**Table 5:** Tooth extraction according to procedures and tooth groups

	**Simple tooth extraction**	**Surgical tooth extraction**	**Retained tooth extraction**	**Total**
Incisors	2,834 (81%)	650 (19%)	1 (0%)	3485
Canines	1,328 (62%)	798 (37%)	8 (0%)	2134
Premolars	4,655 (61%)	2,939 (39%)	14 (0%)	7,608
1^st^ molars	5,709 (44%)	7,211 (56%)	5 (0%)	12,925
2^nd^ and 3^rd^ molars	4,266 (51%)	2,598 (31%)	1421 (17%)	8285
Total	18,792 (54.6%)	14,196 (41.2%)	1,449 (4.2%)	34,437

### Antibiotic prescription according to type of tooth extraction

For this analysis we used data from patients with single tooth extractions (dataset-single-extractions). Neither prescription rates nor antibiotic type differed between men and women or were influenced by endocarditis risk or by bisphosphonate co-medication.

A simple tooth extraction was done in 2.8%, 3.2%, 5.5%, 6.4%, and 3.5% in incisors, canines, premolars, 1^st^ and 2^nd^ molars, and 3^rd^ molars, respectively. A surgical tooth extraction was done in 27.2%, 7.1%, 9.8%, 12.8%, and 14.2% in incisors, canines, premolars, 1^st^ and 2^nd^ molars, and 3^rd^ molars, respectively. Table 6 presents antibiotic prescription according to type of tooth extraction. Antibiotic prescription trend increased from simple to surgical and retained tooth extraction (χ^2^ = 185; *p* < 0.001). Antibiotic prescription was highest in patients with retained 3^rd^ molars tooth extraction.

**Table 6:** Antibiotic prescription according to type of tooth extraction

		**Patients without antibiotic treatment**	**Patients with antibiotic treatment**	**ODDS ratio**	**OR ratio**^a^
**Simple tooth extraction**	incisors	2,755	79	0.029	1
	canines	1,286	42	0.033	1.14
	premolar	4,397	258	0.059	2.02
	1^st^ and 2^nd^ molars	5,344	365	0.068	2.36
	3^rd^ molars	4,116	150	0.036	1.26
**Retained tooth extraction**	incisors	1	0	-	-
	canines	5	3	-	-
	premolar	11	3	-	-
	1^st^ and 2^nd^ molars	4	1	-	-
	3^rd^ molars	1,010	411	0.407	14.03
**Surgical tooth extraction**	incisors	603	47	0.078	2.69
	canines	741	57	0.077	2.65
	premolar	2,652	287	0.108	3.73
	1^st^ and 2^nd^ molars	6,290	921	0.146	5.05
	3^rd^ molars	2,230	368	0.165	5.69

^a^vs. extracted incisors, number/cell > 10

Although the number of medical procedure and tooth group extraction did not differ between dentists and TMM-SURGEON, dentists significantly more often prescribed antibiotic medicines compared to TMM-SURGEON for simple as well as for surgical extraction, but not when extracting a retained tooth.

## Discussion

This retrospective cohort study utilized a large-scale dataset to evaluate patterns of antibiotic prescription after tooth extraction and factors affecting antibiotic prescription from a regional health insurance in Austria. We found that about 10% of patients undergoing tooth extraction received antibiotic medicine, which is similar to reports from studies conducted in the UK [33] and Germany [5]. In Belgium, however, only 4.2% of patients had an antibiotic prescription after dental procedures [4]. In the Belgian sample the small number of patients under study and the 2-week self-reporting period may contribute to this difference.

Our second finding was that 0.3% (157) of patients with tooth extractions had an endocarditis risk, from which only 8 patients received an antibiotic therapy. The reported efficacy of prophylactic antibiotics to prevent or minimize bacteremia has been discussed controversially [34, 35]. Also, the existing evidence and current guideline recommendation do not support the extensive use of antibiotics [36–38]. A recent study showed that dentists routinely prescribe antibiotics to avoid posttreatment complications or even for nonclinical factors (e.g. patients’ insistence or postponing procedures) [14]. However, the percentage of antibiotic use in this population at risk was smaller than that in the population at large. This suggest that a symptomatic risk screening has not been conducted on the basis of underlying concomitant diseases, but prescription of medications was predominantly guided by physician’s perception.

Thirdly, our results showed that 2.1% of patients with tooth extraction had a prescription for a bisphosphate. Bisphosphonate can induce medication-related osteonecrosis of the jaw, and is associated with osteonecrosis after dental extraction in patients with osteoporosis ranging from 0.09% to 0.34% [39]. The link between osteonecrosis of the jaw and bisphosphonate intake is well-known and the benefit of antibiotic prophylaxis and/or treatment in these patients has been discussed in previous studies [40, 41].

Furthermore, the rate of antibiotic prescription was higher in female than in male patients. Also, younger age was associated with higher rates of antibiotic prescription. This is likely because complex procedures, which involve invasive techniques to extract impacted teeth are more often done in younger patients, while older patients tend to have simple tooth extractions due to periodontal diseases [42, 43].

Comparison of antibiotic use between surgeons (TMM-surgeon), dentists, and dental clinics (DOC) showed that antibiotics were more often prescribed by dentists in both single and multiple teeth extraction. While the most frequent antibiotic prescribed by dentists and DOC was amoxicillin/clavulanic acid, TMM-surgeons prescribed preferentially clindamycin. It should be noted that before 1989 dentists completed their studies in general medicine and then started their specialist training for dental, oral and maxillofacial medicine in Austria. Since 1998 dentists in Austria have established a separate curriculum, with the exception of orthodontics. A previous study in Austria found that only 6.6% of dentists completed special training on antibiotics and 85.2% graduated the compulsory advanced training diploma of the Austrian Dental Association (ÖZÄK). However, there was no association between the frequency of antibiotic prescription in relation to training and duration of practice [44]. In contrast, a study from Asia reported a significant difference in the likeliness of antibiotic prescribing and the level of qualification, which may be linked to different education systems [45].

Furthermore, our results showed that the most frequently prescribed antibiotics after tooth extraction were clindamycin and amoxicillin/clavulanic acid. Patient-reported penicillin allergy is a common reason why dentists may prescribe clindamycin in Austria. Penicillin allergy occurs in 10–20% of patients, yet a high percentage of patient-reported history of penicillin allergy are likely to be erroneous [46]. While penicillin with or without beta-lactamase inhibitors are the most frequently prescribed antibiotics by dentists, clindamycin prescription varies among different countries [5, 14, 47–50]. These differences are likely due to different study design, demographics, health policies, and clinical experiences. Furthermore, the present study investigated not only single but also multiple teeth extractions on the index day, which may result in a greater frequency of broad-spectrum antibiotic prescription as a precautionary measure.

Resistance to antibiotics is still a concerning issue in patients with valvular heart disease and the AHA/ACC guidelines have a clear recommendation when to prescribe these medicines [51]. The indication for prophylactic antibiotic treatment is therefore limited as most periodontal and dental diseases are best managed by simple oral hygiene measures and operative interventions. However, as shown in our study dentists empirically prescribe broad spectrum antibiotics. Recent studies may offer new innovative therapies as a possible alternative [52]. Hence, educational programs for dentist and patients should be promoted. In Sweden, such a program achieved a reduction in the prescription rate of antibiotic prescription [53].

Our study has several limitations. Besides the retrospective design, the lack of information on dosage, frequency and duration of administration, the combinations of antibiotics, and the reasons for individual prescriptions have to be mentioned. Furthermore, we did not have data on clinical course and patient’s outcome after taking antibiotic medicine.

## Conclusions

In conclusion, the general adherence to guideline recommendations for antibiotic prescription after tooth extractions is low. Prophylactic antibiotic prescription is not limited to high-risk patients. Hence, a training program to improve antibiotic use in dental practice to selected patients at risk rather than to reduce clinical symptoms such as pain and swelling should be considered.

## Data Availability

The datasets used and/or analysed during the current study available from the corresponding author on reasonable request.

## Abbreviations

ÖGK: Österreichische Gesundheitskasse
ICD–10: International Classification of Diagnosis
MEL: Medical interventions and services
ATC: Anatomical Therapeutic Chemicals
TMM-SURGEON: Tooth/Mouth/Maxillo-surgeon
Dentists: Dental practitioner
DOC: TMM-SURGEON/dentists in out-patient clinics
SD: Standard deviation
ÖZÄK: Austrian Dental Association
amoxi/clav: Amoxicillin/clavulanic acid

## References

[1] Dar-Odeh NS, Abu-Hammad OA, Al-Omiri MK (2010). Antibiotic prescribing practices by dentists: a review. Ther Clin Risk Manag.

[2] Lodi G, Azzi L, Varoni EM (2021). Antibiotics to prevent complications following tooth extractions. Cochrane Database Syst Rev.

[3] Vlcek D, Razavi A, Kuttenberger JJ (2014). Antibiotics in third molar surgery. Swiss Dent J.

[4] Mainjot A, D'Hoore W, Vanheusden A (2009). Antibiotic prescribing in dental practice in Belgium. Int Endod J.

[5] Halling F, Neff A, Heymann P (2017). Trends in antibiotic prescribing by dental practitioners in Germany. J Craniomaxillofac Surg.

[6] Roberts RM, Bartoces M, Thompson SE (2017). Antibiotic prescribing by general dentists in the United States, 2013. J Am Dent Assoc.

[7] Jaunay T, Sambrook P, Goss A (2000). Antibiotic prescribing practices by South Australian general dental practitioners. Aust Dent J.

[8] Preus HR, Fredriksen KW, Vogsland AE (2017). Antibiotic-prescribing habits among Norwegian dentists: a survey over 25 years (1990–2015). Eur J Oral Sci.

[9] Chiu WY, Yang WS, Chien JY (2018). The influence of alendronate and tooth extraction on the incidence of osteonecrosis of the jaw among osteoporotic subjects. PLoS One.

[10] Otto S, Troltzsch M, Jambrovic V (2015). Tooth extraction in patients receiving oral or intravenous bisphosphonate administration: A trigger for BRONJ development?. J Craniomaxillofac Surg.

[11] Ruggiero SL, Dodson TB, Fantasia J (2014). American Association of Oral and Maxillofacial Surgeons position paper on medication-related osteonecrosis of the jaw–2014 update. J Oral Maxillofac Surg.

[12] Scoletta M, Arata V, Arduino PG (2013). Tooth extractions in intravenous bisphosphonate-treated patients: a refined protocol. J Oral Maxillofac Surg.

[13] Global burden of bacterial antimicrobial resistance in 2019: a systematic analysis. Lancet. 2022;399:629–55. 10.1016/s0140-6736(21)02724-0. 2022/01/24. 10.1016/S0140-6736(21)02724-0 PMC8841637 35065702

[14] Karobari MI, Khijmatgar S, Bhandary R (2021). A Multicultural Demographic Study to Analyze Antibiotic Prescription Practices and the Need for Continuing Education in Dentistry. Biomed Res Int.

[15] Palmer N (2016). Antibiotic prescribing: Embrace antimicrobial stewardship. Br Dent J.

[16] Singh Gill A, Morrissey H, Rahman A. A Systematic Review and Meta-Analysis Evaluating Antibiotic Prophylaxis in Dental Implants and Extraction Procedures. Medicina (Kaunas). 2018;54(6):95. 10.3390/medicina54060095. 2018/12/06. 10.3390/medicina54060095 PMC6306745 30513764

[17] Sweeney LC, Dave J, Chambers PA (2004). Antibiotic resistance in general dental practice”a cause for concern?. J Antimicrob Chemother.

[18] Weist K, Högberg LD. ECDC publishes 2015 surveillance data on antimicrobial resistance and antimicrobial consumption in Europe. Euro Surveill. 2016;21(46):30401. 10.2807/1560-7917.Es.2016.21.46.30399. 2016/12/06. 10.2807/1560-7917.ES.2016.21.46.30399 PMC5144945 27918266

[19] Kirchner S, Springer B, Su Y, Fuchs R, Fuchs K, Reisenzein H, Persen U, Allerberger F (2017). Use of antibiotics in Austria / Antibiotikaeinsatz in Österreich Die Bodenkultur. Journal of Land Management, Food and Environment.

[20] Gutema G, Ali S, Suleman S (2021). Trends of community-based systemic antibiotic consumption: Comparative analyses of data from Ethiopia and Norway calls for public health policy actions. PLoS One.

[21] WHO Regional Office for Europe Antimicrobial Medicines Consumption (AMC) Network. AMC data 2011–2017. 2020. https://apps.who.int/iris/bitstream/handle/10665/330466/9789289054744-eng.pdf.

[22] Vandael E, Magerman K, Coenen S, et al. Antibiotic consumption in Belgian acute care hospitals: analysis of the surveillance methodology, consumption evolution 2003 to 2016 and future perspectives. Euro Surveill. 2019;24(46):1900098. 10.2807/1560-7917.Es.2019.24.46.1900098. 2019/11/28. 10.2807/1560-7917.ES.2019.24.46.1900098 PMC6864973 31771707

[23] Habib G, Lancellotti P, Antunes MJ (2015). 2015 ESC Guidelines for the management of infective endocarditis: The Task Force for the Management of Infective Endocarditis of the European Society of Cardiology (ESC). Endorsed by: European Association for Cardio-Thoracic Surgery (EACTS), the European Association of Nuclear Medicine (EANM). Eur Heart J.

[24] Wilson W, Taubert KA, Gewitz M (2008). Prevention of infective endocarditis: guidelines from the American Heart Association: a guideline from the American Heart Association Rheumatic Fever, Endocarditis and Kawasaki Disease Committee, Council on Cardiovascular Disease in the Young, and the Council on Clinical Cardiology, Council on Cardiovascular Surgery and Anesthesia, and the Quality of Care and Outcomes Research Interdisciplinary Working Group. J Am Dent Assoc.

[25] Menon RK, Gopinath D, Li KY (2019). Does the use of amoxicillin/amoxicillin-clavulanic acid in third molar surgery reduce the risk of postoperative infection? A systematic review with meta-analysis. Int J Oral Maxillofac Surg.

[26] Ramos E, Santamaria J, Santamaria G (2016). Do systemic antibiotics prevent dry socket and infection after third molar extraction? A systematic review and meta-analysis. Oral Surg Oral Med Oral Pathol Oral Radiol.

[27] Thornhill MH, Gibson TB, Yoon F (2022). Antibiotic Prophylaxis Against Infective Endocarditis Before Invasive Dental Procedures. J Am Coll Cardiol.

[28] Pasupathy S, Alexander M (2011). Antibiotic prophylaxis in third molar surgery. J Craniofac Surg.

[29] Sekhar CH, Narayanan V, Baig MF (2001). Role of antimicrobials in third molar surgery: prospective, double blind, randomized, placebo-controlled clinical study. Br J Oral Maxillofac Surg.

[30] Hirsch JA, Leslie-Mazwi TM, Nicola GN (2015). The ICD-10 system: a gift that keeps on taking. J Neurointerv Surg.

[31] Mad P, Geiger-Gritsch S, Hinterreiter G (2012). Pre-coverage assessments of new hospital interventions on Austria: methodology and 3 years of experience. Int J Technol Assess Health Care.

[32] Sheikh Rezaei S, Šinkovec H, Schöberl A (2021). Utilization of potentially inappropriate medication and risk of adverse drug events among older adults with chronic renal insufficiency: a population-wide cohort study. BMC Geriatr.

[33] Cope AL, Francis NA, Wood F (2016). Antibiotic prescribing in UK general dental practice: a cross-sectional study. Community Dent Oral Epidemiol.

[34] Hall G, Hedström SA, Heimdahl A (1993). Prophylactic administration of penicillins for endocarditis does not reduce the incidence of postextraction bacteremia. Clin Infect Dis.

[35] Lockhart PB, Brennan MT, Sasser HC (2008). Bacteremia associated with toothbrushing and dental extraction. Circulation.

[36] Vahanian A, Beyersdorf F, Praz F (2021). 2021 ESC/EACTS Guidelines for the management of valvular heart disease: Developed by the Task Force for the management of valvular heart disease of the European Society of Cardiology (ESC) and the European Association for Cardio-Thoracic Surgery (EACTS). Eur Heart J.

[37] Habib G, Lancellotti P, Antunes MJ (2015). 2015 ESC Guidelines for the management of infective endocarditis: The Task Force for the Management of Infective Endocarditis of the European Society of Cardiology (ESC)Endorsed by: European Association for Cardio-Thoracic Surgery (EACTS), the European Association of Nuclear Medicine (EANM). Eur Heart J.

[38] Microbiology EbtESoC, Diseases I, Infection btISoCf (2009). The Task Force on the Prevention, Diagnosis, and Treatment of Infective Endocarditis of the European Society of Cardiology (ESC). Eur Heart J.

[39] Gaudin E, Seidel L, Bacevic M (2015). Occurrence and risk indicators of medication-related osteonecrosis of the jaw after dental extraction: a systematic review and meta-analysis. J Clin Periodontol.

[40] Bermúdez-Bejarano EB, Serrera-Figallo M, Gutiérrez-Corrales A (2017). Prophylaxis and antibiotic therapy in management protocols of patients treated with oral and intravenous bisphosphonates. J Clin Exp Dent.

[41] Gutiérrez JL, Bagán JV, Bascones A (2006). Consensus document on the use of antibiotic prophylaxis in dental surgery and procedures. Med Oral Patol Oral Cir Bucal.

[42] Tonetti MS, Bottenberg P, Conrads G (2017). Dental caries and periodontal diseases in the ageing population: call to action to protect and enhance oral health and well-being as an essential component of healthy ageing – Consensus report of group 4 of the joint EFP/ORCA workshop on the boundaries between caries and periodontal diseases. J Clin Periodontol.

[43] Passarelli PC, Pagnoni S, Piccirillo GB (2020). Reasons for Tooth Extractions and Related Risk Factors in Adult Patients: A Cohort Study. Int J Environ Res Public Health.

[44] Awad S (2018). Evaluierung der Antibiotikaverschreibung durch Zahnärzte in Österreich.

[45] Wasan H, Gupta P, Mathur A (2017). Influence of Qualification and Practice Settings of Dental Practitioners on Antimicrobial Prescribing in Delhi and National Capital Region. India. J Nat Sci Biol Med.

[46] West RM, Smith CJ, Pavitt SH (2019). 'Warning: allergic to penicillin': association between penicillin allergy status in 2.3 million NHS general practice electronic health records, antibiotic prescribing and health outcomes. J Antimicrob Chemother.

[47] Choi YY (2020). Prescription of antibiotics after tooth extraction in adults: a nationwide study in Korea. J Korean Assoc Oral Maxillofac Surg.

[48] Durkin MJ, Hsueh K, Sallah YH (2017). An evaluation of dental antibiotic prescribing practices in the United States. J Am Dent Assoc.

[49] Lalloo R, Solanki G, Ramphoma K, et al. Antibiotic-prescribing patterns of South African dental practitioners following tooth extractions. J Investig Clin Dent. 2017;8(4). 10.1111/jicd.12247. 2016/10/26. 10.1111/jicd.12247 27778471

[50] Pipalova R, Vlcek J, Slezak R (2014). The trends in antibiotic use by general dental practitioners in the Czech Republic (2006–2012). Int Dent J.

[51] Nishimura RA, Otto CM, Bonow RO (2017). 2017 AHA/ACC Focused Update of the 2014 AHA/ACC Guideline for the Management of Patients With Valvular Heart Disease: A Report of the American College of Cardiology/American Heart Association Task Force on Clinical Practice Guidelines. Circulation.

[52] Ramzan M, Karobari MI, Heboyan A (2022). Synthesis of Silver Nanoparticles from Extracts of Wild Ginger (Zingiber zerumbet) with Antibacterial Activity against Selective Multidrug Resistant Oral Bacteria. Molecules.

[53] Lund B, Cederlund A, Hultin M (2020). Effect of governmental strategies on antibiotic prescription in dentistry. Acta Odontol Scand.

